# The Efficacy of Thoracic Ultrasonography in Postoperative Newborn Patients after Cardiac Surgery

**DOI:** 10.21470/1678-9741-2017-0017

**Published:** 2017

**Authors:** Erkut Ozturk, Ibrahim Cansaran Tanidir, Okan Yildiz, Yakup Ergul, Alper Guzeltas

**Affiliations:** 1 Department of Pediatric Cardiology, Istanbul Saglik Bilimleri University Istanbul Mehmet Akif Ersoy Thoracic and Cardiovascular Surgery Education and Research Hospital, Istanbul, Turkey; 2 Istanbul Gelisim University, Istanbul, Turkey; 3 Department of Cardiovascular Surgery, Istanbul Saglik Bilimleri University Istanbul Mehmet Akif Ersoy Thoracic and Cardiovascular Surgery Education and Research Hospital, Istanbul, Turkey

**Keywords:** Pulmonary Atelectasis, Cardiac Surgical Procedures, Infant, Newborn, Postoperative Care, Ultrasonography

## Abstract

**Objective:**

In this study, the efficacy of thoracic ultrasonography during echocardiography was evaluated in newborns.

**Methods:**

Sixty newborns who had undergone pediatric cardiac surgery were successively evaluated between March 1, 2015, and September 1, 2015. Patients were evaluated for effusion, pulmonary atelectasis, and pneumothorax by ultrasonography, and results were compared with X-ray findings.

**Results:**

Sixty percent (n=42) of the cases were male, the median age was 14 days (2-30 days), and the median body weight was 3.3 kg (2.8-4.5 kg). The median RACHS-1 score was 4 (2-6). Atelectasis was demonstrated in 66% (n=40) of the cases. Five of them were determined solely by X-ray, 10 of them only by ultrasonography, and 25 of them by both ultrasonography and X-ray. Pneumothorax was determined in 20% (n=12) of the cases. Excluding one case determined by both methods, all of the 11 cases were diagnosed by X-ray. Pleural effusion was diagnosed in 26% (n=16) of the cases. Four of the cases were demonstrated solely by ultrasonography, three of them solely by X-ray, and nine of the cases by both methods. Pericardial effusion was demonstrated in 10% (n=6) of the cases. Except for one of the cases determined by both methods, five of the cases were diagnosed by ultrasonography. There was a moderate correlation when all pathologies evaluated together (k=0.51).

**Conclusion:**

Thoracic ultrasonography might be a beneficial non-invasive method to evaluate postoperative respiratory problems in newborns who had congenital cardiac surgery.

**Table:** 

Abbreviations, acronyms & symbols	
CT	= Computerized tomography
ICU	= Intensive care unit
NPV	= Negative predictive value
PPV	= Positive predictive value
RACHS-1	= Risk Adjustment for Congenital Heart Surgery-1
USG	= Ultrasonography

## INTRODUCTION

The diagnosis of respiratory complications such as pleural effusion, pulmonary atelectasis, and pneumothorax are important in postoperative management of neonates after cardiac surgery. Imaging techniques are as well used verification of correct positions of chest tubes, central venous lines, and endotracheal tubes. X-ray is the most frequently used method in differential diagnosis of these intrathoracic pathologies, but radiation exposure due to repetitive examination might have hazardous effect for neonates^[1-3]^.

Radiation dose reduction is especially important for newborns, as they are more sensitive to harmful effects of ionizing radiation. Considering the long-life expectancy of newborns risk for development of immune dysfunction, cataract and malignancy increased after radiation exposure. Therefore, every effort should be made to minimize radiation exposure whenever possible, especially in neonatal period^[4]^.

Thorax ultrasonography (USG) is fast, repeatable, and harmless. The use of thorax USG in cardiac intensive care units (ICU) is steadily increasing. However, little data is available concerning the pediatric and neonatal populations^[4,5]^.

In this study, the efficiency of thorax USG during echocardiography was evaluated in postoperative neonates.

## METHODS

Sixty neonates operated on between March 1, 2015, and September 1, 2015, were included in the study. The study was approved by the local ethics committee. A routine chest X-ray was performed on each of the patients every morning after the operation during their ICU stay, along with echocardiography and thorax USG for the evaluation of pleural effusion, pericardial effusion, atelectasis, and pneumothorax. USG and X-ray findings were compared.

**Fig. 1:**
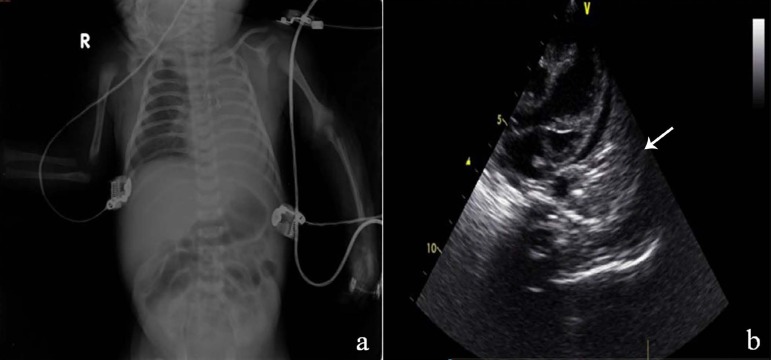
A 19-day old patient, operated for coarctation of aorta, on the 5^th^ postoperative day. A) Chest X-ray revealed increased opacity on the left lung and B) Thorax USG showed lung consolidation with clearly demarcated borders.

Antero-posterior bedside chest radiographs were obtained using portable X-ray equipment. Atelectasis was identified as an essentially homogenous opacity with loss of normal radiolucency. Pleural effusion in the supine position was considered as an increased homogenous density superimposed over lung fields. Pneumothorax was identified by an increased radiolucency without lung markings in the costophrenic angle. Pericardial effusion was suspected when the X-ray showed an enlarged cardiac silhouette with or without an epicardial fat-pad sign and with lungs typically clear (Figure 1A).

Thorax USG was performed by a single pediatric cardiac intensivist (EO) skilled in echocardiographic examinations, using a Vivid S5 (GE, Vivid S5, Norway) with 7-MHz transducers. Examination was performed according to the literature (Figure 1B).

Exclusion criteria included congenital pulmonary problems, premature birth, and neurological problems causing respiratory distress. The data were collected from the medical records of the pediatric cardiovascular surgery ICU. The data reviewed included age, sex, weight, and the type and diagnosis of congenital anomalies. The operative data included lesion and type of repair by Risk Adjustment for Congenital Heart Surgery-1 (RACHS-1) risk category^[6]^.

Statistical analysis was performed using the Statistical Package for Social Science (SPSS, Chicago, Il, USA) version 15.0 for Windows. The descriptive analysis (frequency, median and range) was used to identify the general and specific features of the studied sample. *P*<0.05 was considered statistically significant.

To assess the agreements of thorax ultrasound with chest X-ray, Cohen's kappa coefficient ("k") statistics were used, with k values ≤ 0 as indicating no agreement and 0.01-0.20 as none to slight, 0.21-0.40 as fair, 0.41-0.60 as moderate, 0.61-0.80 as substantial, and 0.81-1.00 as almost perfect agreement^[7]^.

## RESULTS

The majority, 60% (n=36) of the patients were male. Median age was 14 days (2-30 days), and median weight was 3.3 kg (2.8-4.5 kg). Median RACHS-1 score was 4 (2-6). The demographic features are listed in Table 1.

**Table 1:** General clinical characteristics of the cases.

Characteristic	Total (n=60)
Age, day, median (range)	14 (2-30)
Male, n (%)	36 (60)
BMI (kg/m^2^)	0.2 (0.17-0.26)
Weight, kg, median (range)	3.3 (2.8-4.5)
Cardiac Pathology	
HLHS	12
TOF	3
PDA	2
TGA	11
Aortic coarctation	8
TAPVR	5
Truncus arteriosus	3
Pulmonary atresia	7
Complex cardiac pathology	9
RACHS-1	
Undefined	2
2	3
3	17
4	26
6	12

HLHS=hypoplastic left heart syndrome, PDA=patent ductus arteriosus; TGA=transposition of the great arteries; TOF=Tetralogy of Fallot; TAPVD=total abnormal pulmonary venous drainage, RACHS-1=risk adjustment for congenital heart surgery

Atelectasis was demonstrated in 66% (n=40) of the cases. Five of the atelectasis cases were demonstrated only by X-ray, 10 of them by USG, and 25 of them both by X-ray and USG.

Pneumothorax was observed in 20% (n=12) of the cases. While 11 of them were demonstrated only by X-ray, one of them was discerned both by X-ray and USG.

Pleural effusion was demonstrated in 26% (n=16) of the cases. Four of these were demonstrated only by USG, three of them by X-ray, and nine of them both by X-ray and USG.

Pericardial effusion was demonstrated in 10% (n=6) of the cases. While one of the pericardial effusion cases was demonstrated both by X-ray and USG, the remaining five were diagnosed by USG.

Three lung pathologic changes and pericardial effusion were found. There was a moderate correlation between abnormalities detected by thorax USG and X-ray (k=0.51). When thorax USG and X-ray were compared separately for different pathologies, the k was 0.64 for atelectasis, 0.22 for pneumothorax, 0.68 for pleural effusion and 0.28 for pericardial effusion. The evaluation of the presence of atelectasis, pneumothorax, pleural effusion, and pericardial effusion are listed in Table 2.

**Table 2:** The evaluation of chest X-ray and USG of the pathologies.

Observed problem		Only X-ray	Only USG	USG and X-ray
Atelectasis		5	10	25
	Right apical	3	0	2
	Right basal	0	4	6
	Left apical	2	0	3
	Left basal	0	6	14
Pneumothorax		11	-	1
	Right	8	-	0
	Left	3	-	1
Pleural Effusion		3	4	9
	Right	2	2	3
	Left	1	2	6
Pericardial effusion		-	5	1

## DISCUSSION

The benefits of utilizing thorax USG in emergency departments and ICU was supported by multiple studies^[4,8-10]^. In contrast, there is limited data about using thorax USG after congenital heart surgery in newborns. In this study, the efficacy of thorax USG in determining respiratory complications after congenital heart surgery in newborns was compared with that of a chest X-ray. This study's results indicate that thorax USG is both efficient and useful in this condition. This is the first comprehensive study about this topic in the literature to our knowledge.

Atelectasis is a clinical condition, particularly observed frequently in neonates and children who underwent congenital heart surgery. This morbidity can be associated with excessive and viscous pulmonary secretions, and it presents with associated respiratory symptoms. Accumulation of mucus causes susceptibility to pulmonary and systemic infections, causing prolonged need for mechanical ventilation and hospitalization^[11,12]^.

The sensitivity and accuracy of USG in determining consolidation or atelectasis is reported as 80-100% and 90-100%, respectively^[8,13]^. Atelectasis was the most common respiratory complication and was determined in 66% of the cases in the study. Atelectasis was shown in 88% of patients by USG and in 75% by X-ray. Although atelectasis was more frequently diagnosed by USG, imaging of right and left upper lobe atelectasis were difficult. Atelectasis could not be demonstrated in 60% (n=5) of the cases with right upper lobe atelectasis and 40% (n=5) of the cases with left upper lobe atelectasis.

Pneumothorax is defined as the presence of air within the pleural space that prevents full expansion of the lung. Pneumothorax may progress and cause hemodynamic instability, especially in patients receiving positive pressure ventilation^[4,14]^. In the literature, comparing the efficiency of X-ray and USG in determining pneumothorax was found 98% sensitivity, 99% specificity, 98% positive predictive value (PPV), and 99% negative predictive value (NPV) for USG, whereas the results were 75%, 100%, 100%, and 90% for X-ray, respectively^[13]^.

In a study including 126 patients with pneumothorax due to different etiologies (range 2 months-88 years) efficiency of USG in determining pneumothorax was compared with thorax computerized tomography (CT). The sensitivity, specificity, accuracy, PPV, and NPV of chest USG was 89%, 88.5%, 88.9%, 96.7%, and 67.6%, respectively^[15]^. X-ray was more diagnostic than USG for the demonstration of pneumothorax. Only 8% of the cases with pneumothorax (n=12) could be diagnosed by USG. The lack of experience in determining, localizing, and classifying pneumothorax might be the underlying reason in this study.

The other most common pathology after cardiac surgery is pleural effusion^[3,16]^. The compression effect of the effusion leads to different degrees of aeration, even complete loss of alveolar aeration of that particular lung area. Out of the fluid collection, the gradual restoration of the aeration in concordance with gradual manifestation of this consolidative process is often seen. This feature may lead to differentiate the compressive atelectasis from pneumonia^[17]^.

Vezzani et al.^[13]^ demonstrated 100% sensitivity and 99% diagnostic accuracy of chest ultrasound in their series. The false positive result was probably due to the presence of a small pleural effusion not identified by chest X-ray. In this study, pleural effusion was demonstrated in 16 patients; 82% of them were diagnosed by USG and 75% by X-ray.

USG was better than X-ray for the diagnosis of pericardial effusion^[1,4,17]^. Only 16% of the cases (n=1) could be diagnosed by X-ray. Results were similar for both methods for the diagnosis of pleural effusion.

Cost analysis of X-ray and ultrasonography also were compared, resulting in slightly lower costs for ultrasonography, but with a considerable advantage in terms of reduction of ionizing radiation exposure for both patients and staff^[10,17]^. X- ray has certain limitations including risks of radiation exposure, high inter-observer and intra-observer variations. USG is a relatively smaller device that makes point of care more feasible. USG is easy, rapid, portable and repeatable. The USG has also shown less inter and intra-observer variations. Learning curve of techniques and interpretations of USG is simple and fast^[18]^.

The sensitivity, specificity, inter and intra-observer variability were found different in a small number of pediatric studies that compare USG and X-ray to determine the lung pathologies^[5,18]^. Yadav et al.^[19]^ evaluated 118 community-acquired pneumonia cases age between 2-59 months old with X-ray and USG. Abnormal X-ray were found in 101 (85.6%) and abnormal USG in 105 (89%) children. In diagnosing the specific radiological type of pneumonia, very good concordance (Kappa=0.7) was found between X-ray and USG. Kappa was found 0.9 especially in diagnosis of pleural effusion.

In the present study, there was a moderate relation (k=0.51) between USG and X-ray in evaluation of all of the pathologies. The concordance relation was much higher in pleural effusion (k=0.68) and atelectasis (k=0.64) than pneumothorax (k=0.22) and pericardial effusion (k=0.28).

### Limitation

The current study was conducted at a single center with a limited number of cases. It would be more valuable to study this topic in a prospective randomized protocol with a larger patient population. Although computerized tomography (CT) is more accurate and almost a gold standard method in evaluation of thorax pathologies technical difficulties in practice, financial cost and radiation exposure prevent its routine use. Thorax CT might be evaluated in addition to these two modalities.

## CONCLUSION

Thorax USG might be a useful non-invasive method to evaluate the postoperative respiratory problems of neonates after congenital heart surgery. These findings should be supported by further studies.

**Table:** 

Authors' roles & responsibilities	
EO	Conception or design of the work; drafting the work; any part of the work appropriately investigated and resolved; final approval of the version to be published
ICT	Drafting the work; any part of the work appropriately investigated and resolved; final approval of the version to be published
OY	Acquisition and analysis; any part of the work appropriately investigated and resolved; final approval of the version to be published
YE	Revision of the work; any part of the work appropriately investigated and resolved; final approval of the version to be published
AG	Conception or design of the work; revising the work; any part of the work appropriately investigated and resolved; final approval of the version to be published
